# Meiotic restitution mechanisms involved in the formation of *2n* pollen in *Agave tequilana* Weber and *Agave angustifolia* Haw

**DOI:** 10.1186/2193-1801-1-17

**Published:** 2012-09-13

**Authors:** Víctor Manuel Gómez-Rodríguez, Benjamín Rodríguez-Garay, Rodrigo Barba-Gonzalez

**Affiliations:** Centro de Investigación y Asistencia en Tecnología y Diseño del Estado de Jalisco, Unidad de Biotecnología Vegetal, Av. Normalistas No. 800, C.P. 44270 Guadalajara, Jalisco, Mexico

**Keywords:** Agave, Microsporogenesis, Nuclear restitution, Unreduced gametes

## Abstract

A cytological analysis of the microsporogenesis was carried out in the *Agave tequilana* and *A. angustifolia* species. Several abnormalities such as chromosomal bridges, lagging chromosomes, micronuclei, monads, dyads and triads were found. The morphological analysis of the pollen, together with the above-mentioned *2n* microspores, allowed us to confirm the presence of *2n* pollen as well as its frequency. In both *A. tequilana* and *A. angustifolia* two different mechanisms were observed: the first mechanism, a failure in the cytokinesis in meiosis II caused the formation of dyads with two *2n* cells and triads containing two *n* cells and one *2n* cell; the second mechanism, involves an abnormal spindle, which caused the formation of triads with two *n* cells and one *2n* cell. Likewise, the presence of monads was detected in both species, these, might be caused by a failure of the cytokinesis in both meiotic divisions. This is the first report about the presence of a Second Division Restitution mechanism (SDR) which causes the formation of *2n* pollen in the genus *Agave*. The genetic implications of the presence of *2n* pollen in the genus *Agave* are discussed.

## Background

In plants, polyploidy represents an important adaptation and specialization mechanism, and it is estimated that more than 70% of all the angiosperms are polyploids (Ramsey and Schemske 1998; Otto 2007); likewise, molecular analyses suggest that most of the existing angiosperms (>90%) show one or several events of duplication of their genomes, of which many species have undergone this phenomenon only recently (Leitch and Leitch 2008). There are important crops which have some degree of ploidy, such as: cotton, wheat, canola, banana, coffee, tobacco, sugar cane, potato, and many other crops (Udall and Wendel 2006; Brownfield and Köller 2010).

One of the possible origins of the polyploid species is through unreduced gametes (*2n*) (Harlan and De Wet 1975), which occur in most angiosperms (Ramanna and Jacobsen 2003) and have a sporophytic chromosomal number instead of the gametophytic one. These can be formed during the microsporogenesis or megasporogenesis and it has been reported that the production of this type of *2n* gametes is genetically controlled (Mok and Peloquin 1975) by genes which are highly influenced by environmental conditions such as temperature, hydric and nutritional stress (Ramsey and Schemske 1998).

Meiosis is a crucial process in the sexual reproduction of the eukaryotic species, whose purpose is to generate haploid gametes, which includes two successive divisions of the nucleus, where the first division is reductional and the second is equational; the failure of either the first or the second meiotic division leads to the formation of restituted nuclei and therefore the formation of *2n* gametes, however, other possible routes have been proposed, such as: premeiotic failures, abnormal cytokinesis, post-meiotic doubling, the ovule's apomeiotic cells, being the irregular orientations of spindles and abnormal cytokinesis at the second meiotic division the most accepted nowadays (Zhang and Kang 2010). Cytological and genetic studies have proved that there are several mechanisms that are responsible for the formation of this type of *2n* gametes, these mechanisms are known as: First Division Restitution (FDR), Second Division Restitution (SDR) and a third mechanism identified in inter-specific hybrids of the genus *Lilium* known as Indeterminate Meiotic Restitution (IMR) (Lim et al. 2001; Barba-Gonzalez et al. 2005; Zhou et al. 2008; Khan et al. 2010); the effects of the genetic load of these three mechanisms are different because of the different forms of segregation of the chromosomes, since, depending on the present restitution mechanism, the gametes deriving from these will have a different degree of heterozygosity; which means that gametes originated through FDR are identical to one another, maintaining the heterozygosity and the epistasis of their parents; gametes originated through SDR are heterogeneous and have a high homozygosity (Ramanna 1983), and the highest degree of heterogeneity is found in gametes originated through IMR, but since these gametes have a mixture of FDR and SDR, depending on the number of bivalents, the genetic consequences of it are more complex (Lim et al. 2001; Barba-Gonzalez et al. 2008).

Recently, it has been mentioned that in the genus *Agave* some of the species form *2n* pollen (López-Díaz S, personal communication), however, the originating mechanisms have not been elucidated so far, but it is believed that through these gametes the different degrees of ploidy existing in the genus have been generated (Brandham 1969a; Castorena-Sanchez et al. 1991; Ruvalcaba-Ruiz and Rodríguez-Garay 2002; Robert et al. 2008). The genus *Agave* has a basic chromosome number x = 30 and has different degrees of ploidy. Through chromosome counting and flow cytometry, it has been determined that there are chromosomal numbers from 2n = 2x = 60 to 2n = 6x = 180 (Robert et al. 2008); chromosomal studies on the genus have reported it as a bimodal chromosomal model with a constant karyotype, and it has been observed that in the meiosis of *A. tequilana* Weber var. “azul” chromosomes form 30 bivalents (Ruvalcaba-Ruiz and Rodriguez-Garay 2002). Stebbins (1971) observed in this genus the highest degree of chromosomal asymmetry existing in spermatophytes.

The aim of this work was to elucidate the meiotic restitution mechanisms that are involved in the formation of *2n* pollen in *Agave tequilana* and *Agave angustifolia* Haw (2n = *2x* = 60).

In this study, we describe different abnormalities found in the microsporogenesis of *Agave* species, including dyads and triads; further, the morphological analysis of pollen allowed us to confirm the presence of *2n* pollen. Finally, two different restitution mechanisms were identified which are involved in the formation of *2n* gametes.

## Results

### Microsporogenesis analysis and restitution mechanisms

**Figure 1:**
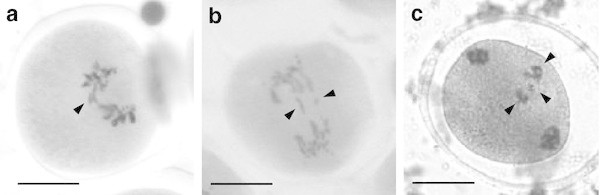
**Meiotic abnormalities found in*A. tequilana*and*A. angustifolia.*a** Formation of a chromosomal bridge and a fragment in the same cell (*arrowhead*). **b** Lagging chromosomes at the center of the cell (*arrowheads*). **c** Three micronuclei with different chromosomal content (*arrowheads*). *Scale bars*: 20 μm.

In the studied species of the genus *Agave*, a successive microsporogenesis, characteristic of this genus, was observed, where immediately after telophase I and telophase II a cytokinesis appears, which causes the formation of a dyad, and finally, a tetrad. During the cytological analysis of the pollen mother cells of *A. tequilana* as well as *A. angustifolia*, several abnormalities were found in both species, such as cells with one or several chromosomal bridges and one or more acentric fragments (Figure 1a), lagging chromosomes (Figure 1b) in both anaphase I and anaphase II as well as micronuclei in telophase I only in *A. angustifolia* var. “Lineño” (Figure 1c), which derived from the lagging chromosomes and/or from the acentric fragments which could not reach the poles and had a different chromosomal content.

**Figure 2:**
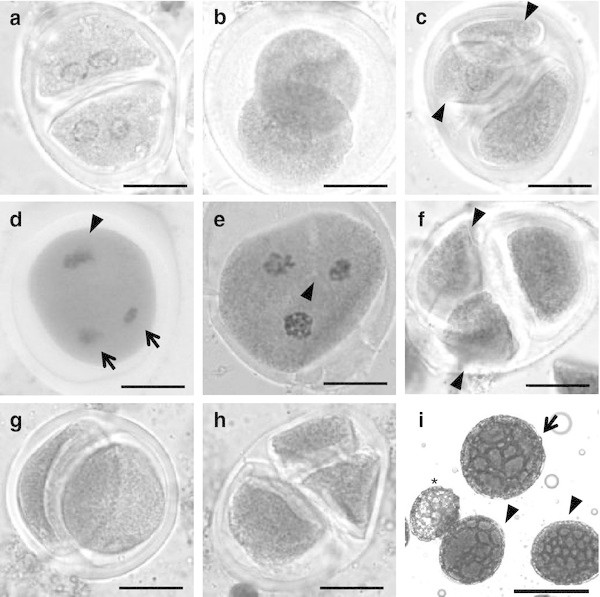
**Meiotic restitution mechanisms in*A. tequilana*and*A. angustifolia.*a** Dyad with two binucleated microspores in *A. angustifolia* var. “Lineño”. **b** Dyad with two *2n* cells in *A. angustifolia* var. “Lineño”. **c** Triad in *A. angustifolia* var. “Lineño”, with two *n* cells (*arrowheads*) and one *2n* cell. **d** Formation of one *2n* restituted nucleus (*arrowhead*) and two *n* nuclei (*arrows*) due to a perpendicular division plane in one of the chromosomal complements in *A. angustifolia* var. “Lineño”. **e** Start of cytokinesis in telophase I (*arrowhead*), the following stage of the one showed in **d**. **f** Triad in *A. angustifolia* var. “Lineño”, with two *n* cells (*arrowheads*) and one *2n* cell in the following stage of e. **g**-**h** Dyad and triad found in *A. tequilana*. **i***2n* pollen (*arrow*), *n* pollen (*arrowheads*) and sterile pollen (*asterisk*) in *A. tequilana*. *Scale bars*: **a**-**h** 20 μm; **i** 100 μm.

In addition to these abnormalities, the presence of monads, dyads and triads could be observed as well as other products of meiosis. Monads and triads were found in both species while no dyads were observed in *A. angustifolia* var. “Cimarron”. The frequencies of monads and dyads were higher in *A. angustifolia* var. “Lineño”, while the frequency of triads was higher in *A. angustifolia* var. “Cimarron” (Table 1). Later on, during the analysis of the meiosis, the origin of the monads, dyads and triads was analyzed. In the cytological analysis of *A. tequilana* and *A. angustifolia* var. “Lineño”, some cells showed a normal first meiotic division, however, a failure in cytokinesis occurred in the second division which generated binucleated dyads (Figure 2a), which would later restitute and give rise to the formation of dyads and triads (Figure 2b-c); dyads were generated because of the absence of cytokinesis in both cells of the dyads, while triads were generated because of the absence of cytokinesis in one of the dyads cells.

**Table 1:** **Observed frequencies of tetrads, triads, dyads and*2n*pollen in two*Agave*species**

Species	Total	Tetrads (%)	Triads (%)	Dyads (%)	Monads (%)	***2n***Pollen (%)
*A. tequilana*	1758	1688 (96)	18 (1)	34 (2)	18 (1)	3.2
*A. angustifolia* var. “Lineño”	3430	3274 (95.5)	8 (0.2)	104 (3)	44 (1.3)	2.0
*A. angustifolia* var. “Cimarrón”	736	725 (98.5)	8 (1.1)	-	3 (0.4)	1.2

Another mechanism of formation of triads in *A. angustifolia* var. “Lineño”, is through the cells which had an abnormal anaphase I, where, once the chromosomes had been reduced, a perpendicular division plane could be observed in one of the chromosomal complements which would be divided equationally (Figure 2d); this caused the formation of two *n* nuclei and one *2n* nucleus in telophase I (Figure 2e); afterwards, the cell would undergo a cytokinesis is, which would cause the formation of a triad (Figure 2f, h). In general, in these species, the frequencies of dyads and triads were 0-3% and 0.2-1% respectively; while the frequency of monads was 0.4-1.3%. The presence of this type of cells indicates the existence of a restitution mechanism in the studied species.

### *2n* pollen occurrence and frequency

Because of the presence of *2n* cells in the cytological analysis, a morphological analysis of the pollen was carried out, which showed the presence of pollen grains of heterogeneous sizes in almost all of the analyzed plants in *A. tequilana*, *A. angustifolia* var. “Lineño” and *A. angustifolia* var. “Cimarron” (Figure 2i). The size of pollen grains varied in both species, in *A. tequilana* a range of 65-139 μm was observed with an average of 93.2 ± 11 μm; for *A. angustifolia* var. “Lineño”, a range of 65-135 μm with an average of 90.9 ± 6 μm; and for *A. angustifolia* var. “Cimarron”, a range of 70-155 μm and an average of 89 ± 7 μm respectively. The frequency of *2n* pollen produced by individual plants ranged from 0-5.8%; in *A. tequilana* there were plants with a percentage of 0-5.8%, and an average of 3.2%; for *A. angustifolia* var. “Lineño”, there were plants with a percentage of 1.1-3.1%, and an average of 2.0%, and for *A. angustifolia* var. “Cimarron” plants with a percentage of 0-2.2% and an average of 1.2% (Table 1).

## Discussion

The analysis of the meiosis in the genus *Agave* began with the studies carried by McKelvey and Sax (1933) and since then, several abnormalities found in the genus have been described (Vignoli 1936; Doughty 1936; Cave 1964; Brandham 1969a). The presence of chromosomal bridges in *A. tequilana* and *A. angustifolia*, have also been reported in other species of the genus *Agave* (Doughty 1936; Cave 1964; Brandham 1969a; Ruvalcaba-Ruiz and Rodriguez-Garay 2002). These bridges are caused mainly by heterozygous paracentric inversions (Brandham 1969b), or they are also caused by the breaking and fusion of chromatids (Newman 1966). Wang et al. (2010) mentioned that irregularities in chromosome pairing contribute to the production of lagging chromosomes and micronuclei; however, the absence of lagging chromosomes in *A. tequilana* and *A. angustifolia* var. “Cimarron” can be interpreted as that the lagging chromosomes joined the other chromosomes at the poles by the end of the anaphase, unlike *A. angustifolia* var. “Lineño”, where micronuclei with different chromosomal content were observed, which were derived from lagging chromosomes or from the breaking of the bridges at different points (Imery-Buiza 2007).

In *A. angustifolia* var. “Lineño”, cells showing an abnormal anaphase I were observed, and after the reductional division of the bivalents, and the separation of the homologous chromosomes, an abnormal spindle in one of the chromosomal complements appeared, which gave rise to a three nucleated cells in telophase I; after that, a cytokinesis is would take place, causing the formation of a triad. The presence of an abnormal and consecutive spindle is a very particular fact, since the formation of *2n* microspores through an abnormal spindle is a mechanism that is completely different to the ones reported in many other species and where the abnormal spindles appear at the same time such as *Zea mays* (Clark 1940), *Agropyrum cristatum* (Tai 1970), *Carthamus tinctorius* (Carapetian and Rupert 1977), *Fuchsia* (Tilquin et al. 1984), *Brassica napus*, *Brassica campestris* (de Souza et al. 1999), *Pfaffia glomerata, Pfaffia tuberosa*, (Taschetto and Pagliarini 2003; Jiang et al. 2009). The presence of these abnormalities implies the formation of *2n* gametes and/or polyads; although this type of spindles has also been found in both meiosis I and meiosis II (Risso-Pascotto et al. 2005).

Failure in cytokinesis is may give rise to nuclear restitution and therefore, the formation of *2n* gametes. The absence of cytokinesis in *A. tequilana* and *A. angustifolia* var. “Lineño” in the second meiotic division caused the formation of dyads and triads, if the absence of cytokinesis was present in both cells of the dyad, then two restituted nuclei were generated and therefore, two *2n* gametes; however, if the failure was present in only one of the cells of the dyad then a triad with two *n* gametes and one *2n* gamete was produced. *2n* microspores resulting from the absence of cytokinesis have been reported in several genera, such as *Paspalum* and *Brachiaria* (Pagliarini et al. 1999; Boldrini et al. 2006), *Solanum* spp. (Werner and Peloquin 1987), *Lolium* (Wagenvoort and Den Nijs 1992) and *Medicago* (Pfeiffer and Bingham 1983), where the failure in cytokinesis was present in both the first and second meiotic divisions, thus generating dyads and triads.

The occurrence of *2n* pollen in a population can be identified by the presence of pollen 1.2-1.3 times larger than normal size, the presence of dyads and triads in the tetrad stage and the unexpected ploidy levels in the progeny from tetraploid-diploid crosses (Veilleux 1985). The occurrence of giant pollen found in this work, as well as the presence of dyads and triads, gave us an indication that the giant observed pollen, is *2n* pollen, which is in accordance with López-Díaz S (personal communication). The morphological analysis of the pollen is the most direct method to determine the presence of *2n* pollen, since by increasing the DNA content of a cell, then its size is larger (Bretagnolle and Thompson 1995); this method has been used as an indicator of the presence of *2n* pollen in several species (Quinn et al. 1974; Ramanna 1983; Sala et al. 1989; Orjeda et al. 1990; Crespel et al. 2006).

Frequencies of *2n* pollen of 1.2-3.2% are reported in this work, however, it was also observed that the frequency in the production of *2n* pollen in individual plants varied, and even in some plants no *2n* pollen was found; this fact has been reported in a large amount of works on diverse genera and species (*Lolium perenne*, Sala et al. 1989; *Ipomoea trifida,* Orjeda et al. 1990; *Ipomoea batatas*, Becerra Lopez-Lavalle and Orjeda 2002; *Pfaffia glomerata, P. tuberosa*, Taschetto and Pagliarini 2003; *Populus tomentosa* Carr, Zhang and Kang 2010), and it has also been reported that variation among individuals can be found within a single taxonomic group (Veilleux 1985) or even from one flower to another in an individual plant, or from one anther to another in the same flower bud (Bretagnolle and Thompson 1995). The identification of the mechanisms that generate *2n* gametes is a complex subject, since different organisms of the same species can produce this kind of gametes through different mechanisms, and more than one mechanism can be present in the same individual plant (Parrot and Smith 1984; Werner and Peloquin 1991; Becerra Lopez-Lavalle and Orjeda 2002; Xu et al. 2008; Zhang and Kang 2010; Wang et al. 2010). A great variety of cytological mechanisms which are involved in the production of *2n* gametes have been found. Bretagnolle and Thompson (1995) reported that the most frequent mechanisms are: failure of spindle in metaphase I or II, an abnormal geometry of the spindle in the second division, and an abnormal cytokinesis. The presence of an abnormal spindle after the chromosomal reduction in *A. angustifolia* var. “Lineño” and the failure in the cytokinesis in *A. tequilana* and *A. angustifolia* var. “Lineño” suggests the presence of a restitution mechanism of the second meiotic division (SDR), since the first meiotic division occurs in a normal way, and it is in the second division where such abnormalities are found. According to Mendiburu and Peloquin (1977) *2n* gametes that are formed by FDR would theoretically transmit 80% of the heterozygosity from the parents to the progeny, and those formed by SDR would only transmit 40%; the reason for this is that in a SDR mechanism the gamete has the two sister chromatids, then, the progeny will have a lesser degree of heterozygosity and epistasis than the parents (Bretagnolle and Thompson 1995). Harlan and De Wet (1975) suggested that sexual polyploidization is the main mechanism in the origin and evolution of polyploids, so these facts corroborate that the evolution of the genus *Agave* may have occurred through *2n* gametes, just as the existence of several degrees of ploidy. Although there are many known polyploid species so far, the reports about the presence of *2n* pollen are much less; in the order Asparagales there are very few reports about the presence of *2n* pollen, some examples are *Asparagus* and *Narcissus* (Camadro 1994; Wu et al. 2011); this is the first report of its kind in the Agavaceae family in which the presence of *2n* pollen, its frequencies, as well as the mechanism through which this type of gametes are formed in *A. tequilana* and *A. angustifolia* are reported.

## Conclusions

Nowadays, commercially important species of *Agave* propagate through suckers and clones obtained through tissue culture, resulting in lower variability compared with the propagation by seeds; the researchers have focused on identifying such variability according to the propagation mechanism involved in the cultivation, in order to develop programs of rehabilitation and conservation of genetic resources in the genus. However, an important alternative to these programs is the use of *2n* pollen, whose presence has been reported in here and in many other genera, and its usefulness in breeding programs has been proven in several crops such potato (Mok and Peloquin 1975), rose (Crespel et al. 2006), lily (Barba-Gonzalez et al. 2004; Lim et al. 2004) and alfalfa (Tavoletti et al. 1991) among others. The importance of this report showing the presence of *2n* pollen and the identification of the restitution mechanisms involved in *2n* gamete formation in *Agave* species, provide basic knowledge for the development of crop improvement programs in the genus, as well as the theoretical effects of the mechanisms involved in the formation of these for *Agave tequilana* var. “azul” and gametes.

## Methods

### Plant material

Flower buds were collected in the Tequila Denomination of Origin Zone for *Agave tequilana* var. “azul” and in Toliman, Jalisco for *Agave angustifolia* var. “Lineño” and *Agave angustifolia* var. “Cimarron”. Voucher specimens for all collections were kept in Unidad de Biotecnología Vegetal at the Centro de Investigación y Asistencia en Tecnología y Diseño del Estado de Jalisco A.C. (CIATEJ) Guadalajara, Mexico.

### Microsporogenesis analyses

Flower buds were collected at various developmental stages and kept in ethanol: acetic acid (3:1) for at least 12 hours and stored at -20°C before the microscopic analysis. Flower buds were classified by size to determine the meiotic stage in the formation of the pollen. Afterwards, the anthers were extracted from the flower buds kept on the above mentioned fixative and rinsed with bidistilled water for 15 minutes, then they were put on a slide and macerated in one drop of aceto-orcein (1%) and finally covered with a cover slip for its observation under a microscope Leica model DMRA2. In mature buds the number of dyads, triads, tetrads and polyads were counted, and in immature buds, the meiosis process was studied, including abnormalities.

### *2n* pollen occurrence and frequency

The mature pollen was dyed with aceto-orcein (1%) to calculate its average diameter and starting from this, the frequency of *2n* pollen; when the diameter of the pollen grains was 1.2-1.4 times the average size of the *n* pollen, it was considered as *2n* pollen (Quinn et al. 1974). Pollen grains were photographed using an Evolution QEi Camera (Media-Cybernetics) attached to the microscope and the measures of their diameter were taken with an Image-Pro® (Media-Cybernetics) software.

## Abbreviations

FDR: First Division Restitution
IMR: Indeterminate Meiotic Restitution
SDR: Second Division Restitution
CIATEJ: Centro de Investigación y Asistencia en Tecnología y Diseño del Estado de Jalisco A.C
Var: Variety.
